# Advances in Cytoprotective Drug Discovery

**DOI:** 10.3390/molecules28114510

**Published:** 2023-06-02

**Authors:** Ekaterina A. Yurchenko, Dmitry L. Aminin

**Affiliations:** G.B. Elyakov Pacific Institute of Bioorganic Chemistry, Far Eastern Branch of Russian Academy of Sciences, pr. 100-letya Vladivostoku, 159, 690022 Vladivostok, Russia; daminin@piboc.dvo.ru

This Special Issue was announced as a platform for authors studying the isolation and identification of various natural products with cytoprotective effects and those studying cytoprotective synthetic compounds. Ten papers describing the results of in vitro, in vivo and in silico investigations have been published.

Animal models were used to examine the effects of cytoprotective compounds in five of the published papers. Pentacyclic triterpene celastrol was investigated in a study by Liu et al. in a murine cerebral ischemia/reperfusion injury model, which revealed the positive effects of celastrol on the cerebral ischemia/reperfusion injury-induced alteration of sphingolipid and glycerophospholipid metabolism [1].

Amphipterygium adstringens (cuachalalate) containing anacardic acids was studied in Balb/c breast-tumor-bearing female mice, which revealed important myeloprotective effects against 5-fluorouracil- and carboplatin-induced myelosuppression and leukopenia. Moreover, Galot-Linaldi and co-authors reported its antineoplastic effects [2].

A rodent isoproterenol-induced myocardial dysfunction model was used by Asdaq et al. to investigate the cardioprotective properties of aged garlic extract and its active constituent, S-allyl-L-cysteine. The histopathological observations corroborated the biochemical findings, and both confirmed that the investigated substances have synergistic effects, with carvedilol in preventing morphological and physiological changes in the myocardium [3].

The neuroprotective effects of curcumin were studied in a neurotoxic rat model induced by aluminum chloride (AlCl_3_) to mimic the sporadic form of Alzheimer’s disease. ELBini-Dhouib and co-authors reported that curcumin enhances the behavior of AlCl_3_-exposed rats and decreases their oxidative stress and inflammation levels [4]. 

A rodent pentylenetetrazole-induced animal model of epilepsy was used by Firdous and co-authors to study the anti-inflammatory activity and anti-convulsion effect of the n-hexane extract of the *Rosa webbiana* fruit found in chitosan nanoparticles. A positive effect on convulsive behavior, morphological differences, neuronal survival and p-TNF-α and p-NF-κB expressions was observed [5].

Three of the papers reported on cytoprotective compounds, the effects of which were studied in vitro. The differentiation of the receptor activator of nuclear factor kappa-Β ligand (RANKL)-induced RAW264.7 cells into osteoclasts was used for the in vitro examination of the effects of ligustroside and oleoside dimethylester, which are natural product-derived compounds isolated from the *Syringa oblata* subsp. *dilatata* plant. Kim and co-authors reported that the compounds inhibited differentiation without cytotoxicity by inhibiting the phosphorylation of signaling pathways, which play a pivotal role in osteoclast differentiation [6].

The protective effects of five lanostane triterpenoids from the marine sponge *Penares* sp. were examined in a paraquat-induced neuroblastoma Neuro-2a cell model of Parkinson’s disease. The authors reported the positive influence of the compounds on various viability aspects of paraquat-treated neuronal cells, including their effect on Hsp70 levels and neurite growth [7]. 

Moreover, the antioxidant effects of marine fungal compounds against oxidative stress in Neuro-2a cells induced by rotenone and paraquat neurotoxins were investigated in a brief report. The *p*-terphenyl polyketides from *Aspergillus candidus* KMM 4676 and cerebroside flavuside B from *Penicillium islandicum* (=*Talaromyces islandicus*) increased the viability and decreased the reactive oxygen species level in these cells [8].

The cell-free testing of the antioxidant properties of the main components of methanol extracts from *Onosma bourgaei* (Boiss.) and *O. trachytricha* (Boiss.) herbs and the in silico study of their enzyme inhibitory activities were reported by Istifli [9].

Finally, the cryoprotective and cytoprotective agents in the cryopreservation process were reviewed by Marcantonini and co-authors. Natural cryoprotective and cytoprotective agents, such as antifreeze proteins, sugars and natural deep eutectic systems, were compared to synthetic agents, addressing their mechanisms and protection efficacy [10].

Thus, this Special Issue is a collection of papers about various cytoprotective drug discoveries, which can undoubtedly reflect the current state of this field of research.
